# Characterization of peri-infarct zone by cardiac magnetic resonance: validation compared to ex-vivo imaging and post-mortem histology

**DOI:** 10.1186/1532-429X-13-S1-P69

**Published:** 2011-02-02

**Authors:** Otavio R Coelho-Filho, Richard N Mitchell, Lawrence S Lee, Rebecca MW Mitchell, Suyog A Mokashi, Rob J van der Geest, Jan A Schimitto, Frederick Chen, Michael Jerosch-Herold, Raymond Y Kwong

**Affiliations:** 1 Brigham and Women's, Boston, MA, USA; 2 George Washington University, Washington, WA, USA; 3 Leiden University Medical Center, The Netherlands., Leiden, Netherlands

## Background

Assessment of peri infarct zone (PIZ) by late gadolinium enhancement (LGE) is limited by the spatial resolution of invivo LGE in the z direction, resulting in partial volume effect. Although the assessment of infarct size by LGE has been validated, characterization of PIZ has not been validated against histopathological findings.

## Objectives

We seek to characterize the PIZ by LGE in comparison with histology in an ischemia reperfusion model of myocardial infarction (MI).

## Methods

Adult sheep (40kg) were subjected to ischemia reperfusion injury (LAD for 45 min). Six animals underwent invivo CMR 10 days after surgery and were euthanized on the next day for exvivo CMR and histopathological examination. Invivo CMR included: black blood T2 and 2D LGE (in-plane resolution 2mm). Animals were euthanized 10 min after injection of Gadolinium. Heart specimens were sliced and imaged exvivo using a high resolution 3D LGE (isotropic resolution of 0.6mm). Heart specimens were then fixed for histological analysis (HE and Masson trichrome).

## Results

**Figure 1:**
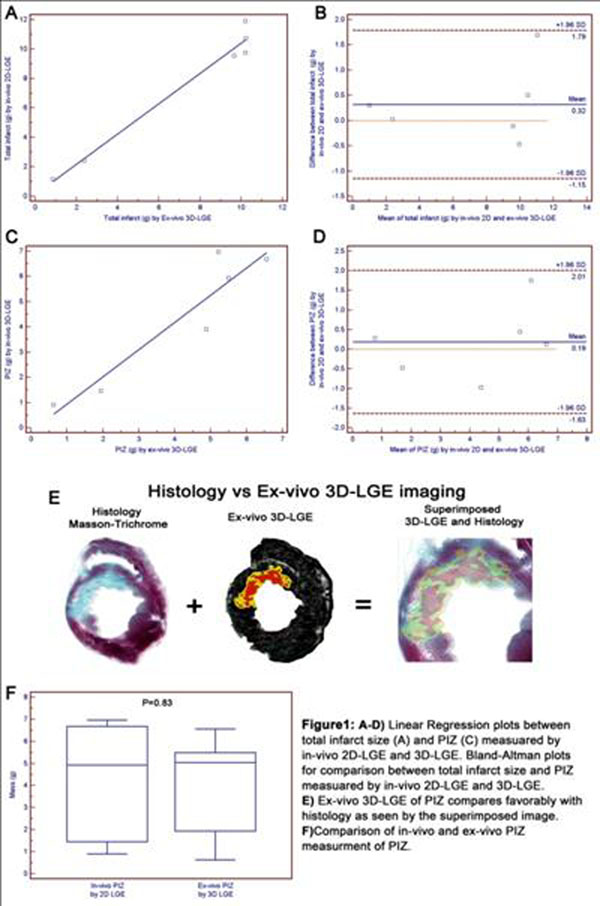
A-D) Linear Regression plots between total infarct size (A) and PIZ (C) measured by in-vivo 2D-LGE and 3D-LGE. Bland-Altman plots for comparison between total infarct size and PIZ measured by in-vivo 2D-LGE and 3D-LGE. E) Ex-vivo 3D-LGE of PIZ compares favorably with histology as seen by the superimposed image. F) Comparison of in-vivo and ex-vivo PIZ measurement of PIZ.

Infarct and PIZ mass were measured of the invivo and exvivo LGE using a previously described criterion (FWH 50%). Invivo MI size (mean 7.58±4.59g) and exvivo MI size (7.26±4.4g) showed strong correlation (R^2^=0.99, p<0.001) and agreement (figure1AB). Invivo (4.31±2.65g) and exvivo PIZ mass (4.12g±2.31g) were not different (p=0.8) (Figure1F) and were strongly correlated (R^2^=0.87, p=0.006, Figure1CD). Morphology and distribution of MI and PIZ (n=6) by exvivo 3D LGE correlated well with matching short axis pathologic sections (Figure1E). Superimposed 3D LGE and pathologic images demonstrated that the PIZ consisted of a layer of viable myocardium, approximately 6-8 myocytes thick.

## Conclusion

CMR imaging of PIZ compares favorably with histology. Clinically available 2D LGE technique provides robust measurement of infarct size and PIZ. The agreement between invivo 2D and exvivo 3D LGE suggests that slice thickness has no relevant effect on PIZ measurement.

